# Fania (Fanny) Kaplan and the attempted assassination of Vladimir Lenin: Ophthalmologic considerations

**DOI:** 10.1111/aos.70073

**Published:** 2026-01-27

**Authors:** Stephen G. Schwartz, Christopher T. Leffler, Peter M. Allen, Andrzej Grzybowski

**Affiliations:** ^1^ Department of Ophthalmology, Bascom Palmer Eye Institute University of Miami Miller School of Medicine Miami Florida USA; ^2^ Department of Ophthalmology Virginia Commonwealth University School of Medicine Richmond Virginia USA; ^3^ Department of Vision and Hearing Sciences Anglia Ruskin University Cambridge UK; ^4^ Department of Ophthalmology University of Warmia and Mazury Olsztyn Poland

**Keywords:** blast‐related injury, ocular trauma, ophthalmic history, visual impairment

## Abstract

**Purpose:**

Fania (Fanny) Kaplan (1890–1918), who was reportedly visually impaired, confessed to the attempted assassination of Soviet leader Vladimir Lenin (1870–1924) in 1918 by shooting him with a pistol. The precise nature of her visual loss is unknown and raises doubts about whether she had sufficient visual function to perform the act.

**Methods:**

Historical documents were reviewed.

**Results:**

The cause of Kaplan's visual loss is uncertain but occurred following a bomb blast in 1906. If the explosion was the cause, then she most likely had bilateral closed‐globe, blast‐related injuries, perhaps with additional functional visual loss. She reportedly received treatment at a medical centre in Kharkov (now Kharkiv), then led by the prominent ophthalmologist Leonard Girshman (1839–1921). An informal estimate of the minimum visual acuity required to shoot an adult at 10 feet (3 m) with a pistol is approximately 1.2 logMAR (Snellen equivalent 20/320 or 6/96).

**Conclusions:**

Based on available historical documents, Kaplan's visual function was most likely sufficient to carry out the assassination attempt, although her visual impairment may have contributed to the attempt being unsuccessful.

## 1 INTRODUCTION

Fania (Fanny) Kaplan (1890–1918), who was reportedly visually impaired, confessed to the attempted assassination of Soviet leader Vladimir Lenin (1870–1924) in 1918 by shooting him with a pistol from about 10 feet (3 m). This event is of historical significance, because it contributed to the Red Terror, a series of reprisals which led to perhaps hundreds of thousands of deaths (Sixsmith, 2017).

## 2 BIOGRAPHIC CONSIDERATIONS

Lenin was shot in Moscow shortly before 10 p.m. on 30 August 1918, while walking past a crowd after delivering a speech. He was hit in the left shoulder and the left side of his neck; a third shot hit a female bystander. Lenin is reported to have survived the neck wound ‘because he had suddenly turned his head at the moment of the shot’ (Lyandres, 1989). Immediately after the shooting, several suspects were arrested, including Kaplan, who was interrogated by the Cheka (secret police) in the notorious Lubyanka prison. She confessed and was executed (Lyandres, 1989). Kaplan, born Feyga Chaimovna Roitblat (alternatively Feiga Khaimovna Roitman), was born into a Jewish family in Volhynia, now in Ukraine (Sixsmith, 2017). She became a revolutionary and began to use the name Fania Kaplan by 1906 (Lyandres, 1989).

On 22 December 1906, Kaplan and an accomplice were assembling or storing one or more bombs in a hotel room in Kiev (now Kyiv) when there was a premature explosion. Kaplan is variously reported to have suffered minor injuries to her extremities and buttocks (Lyandres, 1989) or more severe injuries to her head (Figner, 1930) or her hands and face (Sixsmith, 2017) with subsequent vision and hearing loss. In addition, a hotel chambermaid was fatally wounded. Kaplan was convicted of armed assault and sentenced to death, although the sentence was commuted to life imprisonment in a Siberian katorga, a forced labour camp and a precursor to the gulag (Lyandres, 1989).

Kaplan, while incarcerated, was noted to have at least some visual impairment. Vera Figner (1852–1942), a Russian revolutionary, wrote that ‘her medical report stated that she had gone blind due to hysterical illness’ but that her fellow convicts ‘thought that the head wound [from the bomb blast] had caused her blindness’ (Figner, 1930). Kaplan is reported to have suffered from recurrent episodes, lasting days at a time, with severe headaches and worsened visual loss. She was reported to have become ‘completely blind’ by 1909 (Lyandres, 1989). Figner, quoting a fellow inmate, wrote that ‘a doctor from the regional administration’ examined Kaplan and reported that ‘her pupils were reacting to light and told us to request a transfer to Chita, where she could undergo electrical treatment’ (Figner, 1930). Kaplan attempted suicide at least once but eventually learned Braille and became more independent. She received medical treatment in multiple prison hospitals, including Chita, and her vision was reported to have improved so that she was no longer in the ‘complete darkness in which she had lived for years’ (Lyandres, 1989).

Following the February Revolution in 1917, Kaplan and many other political prisoners were released. There is general agreement that she was hospitalized for further treatment in Kharkov (now Kharkiv); one source reports that she underwent ‘two complex operations’ which ‘succeeded in restoring some of her sight’ (Sixsmith, 2017), but no details were provided. She was subsequently offered a ‘highly paid position in the municipal administration’ in Simferopol, on the Crimean Peninsula, in late 1917, but no details were provided regarding the nature of this employment (Lyandres, 1989). In January 1918, the Bolsheviks took control of Simferopol, and Kaplan lost her job. She arrived in Moscow by March 1918. During the spring of 1918, she was described as ‘a rather pretty woman but undoubtedly demented, and in addition to this with various afflictions: deaf, semi‐blind, in a state of exaltation as if a holy idiot.’ Her subsequent activities in Moscow are unknown until she was arrested following the shooting of Lenin about 5 months later (Lyandres, 1989).

## 3 OPHTHALMOLOGIC CONSIDERATIONS

Some modern historians have questioned whether Kaplan was actually the shooter and have documented many inconsistencies and contradictions within the eyewitness testimonies (Lyandres, 1989). Nevertheless, from an ophthalmologist's perspective, at least two questions are evident. First, what was the nature of her vision loss? And second, what is the minimum level of visual function necessary to shoot an adult with a handgun at 10 feet?

### 3.1 Diagnosis

**FIGURE 1:**
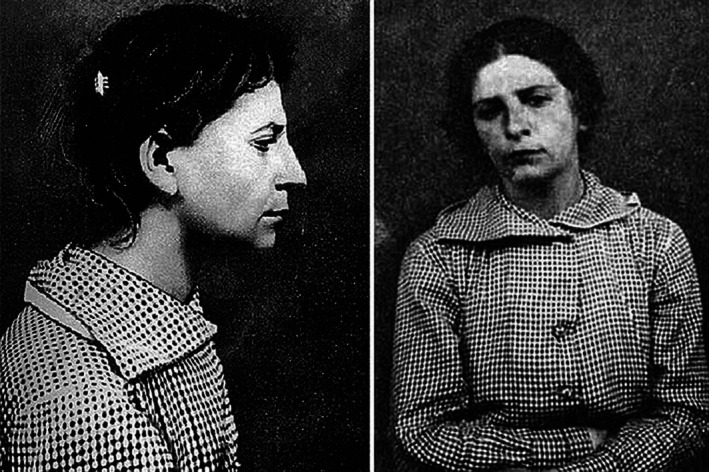
Fania (Fanny) Kaplan, 1918. Image in the public domain.

Establishing a precise diagnosis for Kaplan's visual loss may not be possible. A photograph from 1918 shows apparently normal eyes and no obvious facial scars (Figure 1). The shooting occurred after sundown, and when Kaplan was arrested, she was not wearing eyeglasses (Sixsmith, 2017).

Kaplan was reported by many sources to have suffered visual loss—either from the 1906 bomb explosion or during her subsequent imprisonment—although she later recovered at least ambulatory vision. She is reported to have undergone medical treatments starting in 1912 and possibly eye surgery in 1917. If the cause of the visual loss was the bomb explosion, then this sequence of events is most consistent with bilateral closed globe, blast‐related injuries (Lee et al., 2023). There may have been associated macular injuries, including choroidal rupture or chorioretinitis sclopetaria (Ulianova et al., 2025). In comparison, a review of eye injuries from the September 2024 pagers explosions in Lebanon reported 76 injured eyes of 38 patients; bilateral injuries occurred in 74% of patients and closed globe injuries occurred in 43% of eyes, which is consistent with Kaplan's history (Antoine et al., 2025).

It is unknown if Kaplan underwent eye surgery in Kharkov in 1917, but it is certainly possible, as the Department of Ophthalmology of what is now Kharkiv National Medical University was founded in 1870 by ophthalmologist Leonard Girshman (1839–1921). It seems unlikely that she underwent cataract surgery, for there is no evidence that she wore aphakic glasses following surgery or highly myopic glasses before surgery. Another possibility might be optical iridectomy. A series of 35 cases of optical iridectomy published in 1932 was divided into patients with ‘optical obstruction’ caused by opacities of the cornea or lens versus ‘pupillary deformation’ caused by synechiae (Kaplan was reported to have ‘reactive pupils’ while she was imprisoned, so the latter situation seems less likely). In this series, 9 of 35 patients experienced ‘varying degrees of permanent improvement’ (Foster, 1932).

It is more difficult to explain the nature of, and subsequent recovery from, her ‘complete’ and ‘total’ vision loss. Recovery from no light perception is reported rarely in patients following prompt medical treatment of some optic neuropathies (Thurtell & Kardon, 2012) or surgical repair of open globe trauma (Baban et al., 2015), but this recovery generally happens within days. If Kaplan had reactive pupils in 1912, then it seems likely that she had (at least) light perception, although another possibility is transient cortical blindness (with preserved pupillary reaction) associated with head trauma (Ng, 2016).

The fluctuating nature of her visual impairment, perhaps according to her environment, is notable but unfortunately there are no specifics available. Transient bilateral severe visual loss associated with headaches may be noted in patients with migraine or demyelinating diseases (Uhthoff phenomenon) (Loffler et al., 2025), but again the duration of symptoms would most likely be measured in days. Recurrent intraocular diseases, such as uveitis or intraocular pressure spikes, would be expected to have an overall progressive course; Kaplan, even with the best medical care available at the time, would be expected to have more severe residual visual deficits.

The overall course of Kaplan's disease, specifically ‘total’ blindness lasting for years, followed by recovery, suggests at least some level of functional visual loss. For example, a recent review of studies on functional visual loss reported that 42% of adult patients had a history of psychiatric disorders and 31% reported antecedent physical trauma (Brady et al., 2025), again consistent with Kaplan's history. If she was, in fact, offered an unspecified ‘highly paid position in the municipal administration’ in Simferopol in 1917, her visual recovery must have been substantial.

### 3.2 Necessary visual function for shooting

Regardless of her ophthalmologic diagnosis, what level of visual function would have been necessary to commit the assassination attempt? There is more information in the peer‐reviewed literature on rifle shooting than on pistol shooting. For example, in a prospective trial, ‘elite able‐sighted shooters’ were fitted with Cambridge simulation glasses (to simulate visual impairment) and asked to shoot a rifle at a target 10 m (about 33 feet) away. Based on these results, the investigators proposed that a combined visual acuity of 0.53 logMAR (Snellen equivalent 20/68 or 6/20) and contrast sensitivity 0.83 logCS (testing range, 0–2.25 logCS) would be the level where the athletes with vision impairment are unable to compete in the normal version of the sport (without adaptations such as an auditory signal to help with aim) (Allen et al., 2016).

Unfortunately, there appears to be no analogous peer‐reviewed literature on pistol shooting with visual impairment. A rifle is inherently more accurate than a pistol, because it can be braced against the shoulder for greater stability. In addition, the longer barrel length of a rifle allows a higher bullet velocity than an equivalent bullet fired from a pistol with a shorter barrel. Nevertheless, a motivated shooter of moderate skill could probably hit an adult‐sized target at 3 m by ‘point shooting’ without using the pistol sights. An estimate of the minimum visual acuity required, based on informal experiments in dim illumination, is approximately 1.2 logMAR (Snellen equivalent 20/320 or 6/96). In real‐life situations, there is the added variable of the difference in contrast between the target and background. This is particularly pertinent for people with vision impairment. A potential mitigating factor is if Lenin were talking, because the auditory stimulus might compensate for visual loss. On the other hand, the shooting occurred after nightfall (although it is uncertain how much artificial lighting was available at the location) and while Lenin was walking, which would have increased the difficulty of the shot.

There is no available evidence regarding Kaplan's pistol shooting skills, or hand grip strength, or if she ever received any training. Nevertheless, she reportedly hit Lenin with two of three shots, including a neck wound in which the bullet must have passed within inches of the carotid artery, jugular vein and spinal cord. This seems to compare favourably with statistics from trained police officers using modern weapons and ammunition. For example, one review reported ‘hit rates’ between 18% and 54% of police shootings in the United States in the 21st century. The investigators reported that accuracy is increased by physical fitness, including grip strength and worsened by anxiety (although this is amenable to training) (Simas et al., 2022). The assassination attempt was very nearly successful, which would have had unknowable but potentially profound historical consequences.

## 4 CONCLUSION

Based on the available historical evidence, Kaplan most likely had at least some degree of visual loss. If she was, in fact, the shooter, then it is reasonable to suppose that her visual loss may have contributed to the unsuccessful nature of the assassination attempt.

## FUNDING INFORMATION

Funding/Support: Partially supported by Research to Prevent Blindness Unrestricted Grant GR004596‐1.
